# Initial development and usability testing of a digital fertility preservation navigator for newly diagnosed young adult cancer survivors

**DOI:** 10.1007/s00520-026-11015-z

**Published:** 2026-07-21

**Authors:** Karly M. Ingram, Stephanie Bunch, Mollie V. Rose, Janet A. Tooze, Milena Duque, David Victorson, Betina Yanez, Kristin N. Smith,, Gwendolyn P. Quinn, David P. Miller, John M. Salsman

**Affiliations:** 1 Department of Psychology, East Carolina University, Greenville, NC USA; 2 Department of Social Sciences and Health Policy, Wake Forest University School of Medicine, Winston-Salem, NC USA; 3 Department of Communication, Wake Forest University, Winston-Salem, NC USA; 4 Department of Biostatistics and Data Science, Wake Forest University School of Medicine, Winston-Salem, NC USA; 5 Atrium Health Wake Forest Baptist, Winston-Salem, NC USA; 6 Department of Medical Social Sciences, Northwestern University Feinberg School of Medicine, Chicago, IL USA; 7 Robert H. Lurie Comprehensive Cancer Center, Northwestern University Feinberg School of Medicine, Chicago, IL USA; 8 Department of Obstetrics & Gynecology, NYU Grossman School of Medicine, New York, NY USA; 9 Department of Implementation Science, Wake Forest University School of Medicine, Winston-Salem, NC USA

**Keywords:** Young adult, Antineoplastic protocols, Fertility, Decision support techniques, Digital technology

## Abstract

**Purpose:**

Young adults with cancer (YAs) are rarely provided the necessary resources to make informed decisions about fertility preservation (FP) prior to beginning treatment. Digital decision aids are a promising strategy to ensure YAs receive timely, guideline-concordant information. We developed an alpha version Fertilit-*e*, a digital FP navigator, and conducted usability testing to obtain feedback from YAs.

**Methods:**

We designed an alpha version of Fertilit-*e* that drew from existing evidence-based FP resources. Feedback was obtained from two waves of participants using a think-aloud framework, and modifications were made to the decision aid.

**Results:**

Across waves, participants (*n* = 12) understood the purpose of Fertilit-*e* and thought modules were comprehensive and easy to understand. The usability of Fertilit-e was rated as excellent across waves and devices (iPad and computer). Participants suggested reducing the use of and better defining medical terminology and adding text to videos to enhance comprehensibility.

**Conclusions:**

YAs reported that Fertilit-*e* provided important information regarding FP in an understandable manner. Participants made suggestions for how to improve comprehensibility and usability that will guide future iterations of Fertilit-*e*. For example, tailoring algorithms and reorganization may direct users to the most relevant information.

**Implications for cancer survivors:**

Digital decision aids may be a feasible and useful strategy for helping YAs navigate decisions regarding whether to pursue FP at the time of diagnosis. Additional research is needed to refine and evaluate such tools before integration into routine care.

## Introduction

Every year, approximately 90,0000 adolescents and young adults (AYAs; ages 15–39) are diagnosed with and treated for cancer, which can increase the potential for infertility or other reproductive challenges [1]. The American Society for Reproductive Medicine (ASRM) [2], American Society of Clinical Oncology (ASCO) [3], and the National Comprehensive Cancer Network (NCCN) [4] have established national guidelines to encourage clinicians to discuss fertility preservation (FP) options with newly diagnosed patients prior to proceeding with fertility compromising treatments. These guidelines recommend that (1) oncology clinicians inform all patients about the impact of cancer and/or treatment on their fertility and (2) all patients who wish to receive further information about FP are referred to a reproductive specialist.

Despite these guidelines, many young adults diagnosed with cancer (YAs) are not provided FP information or effective tools for how to best navigate their FP options [5, 6]. Indeed, only half of early-onset patients with cancer reported having a conversation with a healthcare professional about their options to preserve their fertility before beginning treatment [7]. Recent studies suggest that oncology clinicians face many communication challenges when discussing FP with their patients. These challenges can be related to physician attributes (e.g., knowledge and time barriers), patient attributes (e.g., type of cancer, need for immediate treatment, and cultural or religious beliefs), and policy issues (e.g., insurance and referral sources) [8]. In fact, a burgeoning scientific literature on adult oncology clinicians’ practice patterns and barriers to discussing FP suggests that oncologists rarely refer patients to FP specialists [8, 9] with < 25% aware of or distributing written educational materials to their patients [9] and < 50% following recommended ASRM/ASCO/NCCN guidelines [8]. Similar trends hold true for the dearth of FP information and specialized programs that are available to YAs [10]. For example, a recent study found that oncofertility programs were present on only half of comprehensive cancer center websites [11]. Similarly, a survey of National Cancer Institute Community Oncology Research Program practice groups that treat AYAs found that nearly one-third of practices had neither male nor female FP services available, and only half had FP services available for female patients [12].

It is essential that YAs newly diagnosed with cancer receive timely and accurate FP information. Unfortunately, oncofertility patient navigation [13], while highly effective, is not widely available to patients. Expansion of oncofertility care including patient navigation has been limited by a lack of specialized care providers, barriers to funding, and societal beliefs about reproductive healthcare [14]. Given existing deficits in information provision by oncologists and the limited availability of oncofertility patient navigation, alternative channels and strategies may be needed to provide fertility information. Novel patient-facing digital tools are a promising solution. The use of digital interventions has grown significantly in recent years, particularly among YAs [15, 16], increasing the potential to make decision-making interventions more accessible, personally tailored, and integrated into clinical practice [16–18]. By delivering guideline-based FP information to YA patients using digital technologies, patient knowledge may be increased and sustained, and both patients and providers may be empowered to attend to this unaddressed issue through informed decision-making [19]. Indeed, in recent years, there has been a proliferation of such tools that are meant to be used shortly after cancer diagnosis with promising results. For example, Woodard et al. [20] developed a website called Pathways that provides women information about FP options, decision support activities, survivor testimonials, and resources. This website is being evaluated as part of a multicomponent intervention that also includes provider education and telephone counseling in a comparative effectiveness cluster randomized trial [21]. Similarly, Ehrbar et al. have developed and evaluated a digital decision aid for female cancer patients meant to augment fertility counseling that resulted in reductions in decisional conflict and regret [22–24]. Despite this substantial progress, existing fertility-focused interventions for YAs have primarily focused on female patients and are meant to be used in conjunction with other interventions delivered by specialized providers. Thus, additional work is needed to develop self-guided tools that can ensure all YAs have access to FP information and decisional support.

In the present study, we aimed to develop the alpha version of a digital FP navigator, called Fertilit-*e*, designed for male and female patients and that could be used independent of a clinical encounter. Fertilit-*e* would address knowledge deficits and promote informed decision-making about fertility options among YAs newly diagnosed with cancer and their providers. We achieved this goal by adapting existing fertility-preservation content for tailored, rapid, and clear dissemination of information in an engaging, cross-platform, and patient-friendly digital format. We then alpha-tested Fertilit-*e* with end users to evaluate usability and comprehensibility to inform future refinements to content and design.

## Methods

This study recruited YAs from the Atrium Health Wake Forest Baptist Comprehensive Cancer Center (AHWFBCCC). All study procedures described were approved by the Wake Forest University School of Medicine Institutional Review Board (IRB) and performed in accordance with the Declaration of Helsinki. This project was supported by AHWFBCCC’s Cancer Prevention and Control Program pilot award and registered on ClinicalTrials.gov (NCT03599661, registration first submitted 7/13/2018).

### Development of Fertilit-e alpha

The alpha version of Fertilit-e was developed as a secure, web-based application in collaboration with the Research Information Systems Unit (RISU) at AHWFBCCC. For Fertilit-e, the RISU configured the web-based application infrastructure, developed the application and supporting database, implemented study-specific functionality, and provided ongoing technical support. This collaboration allowed the study team to translate the intervention framework, tailoring logic, and fertility preservation content into an accessible online tool.

**Fig. 1:**
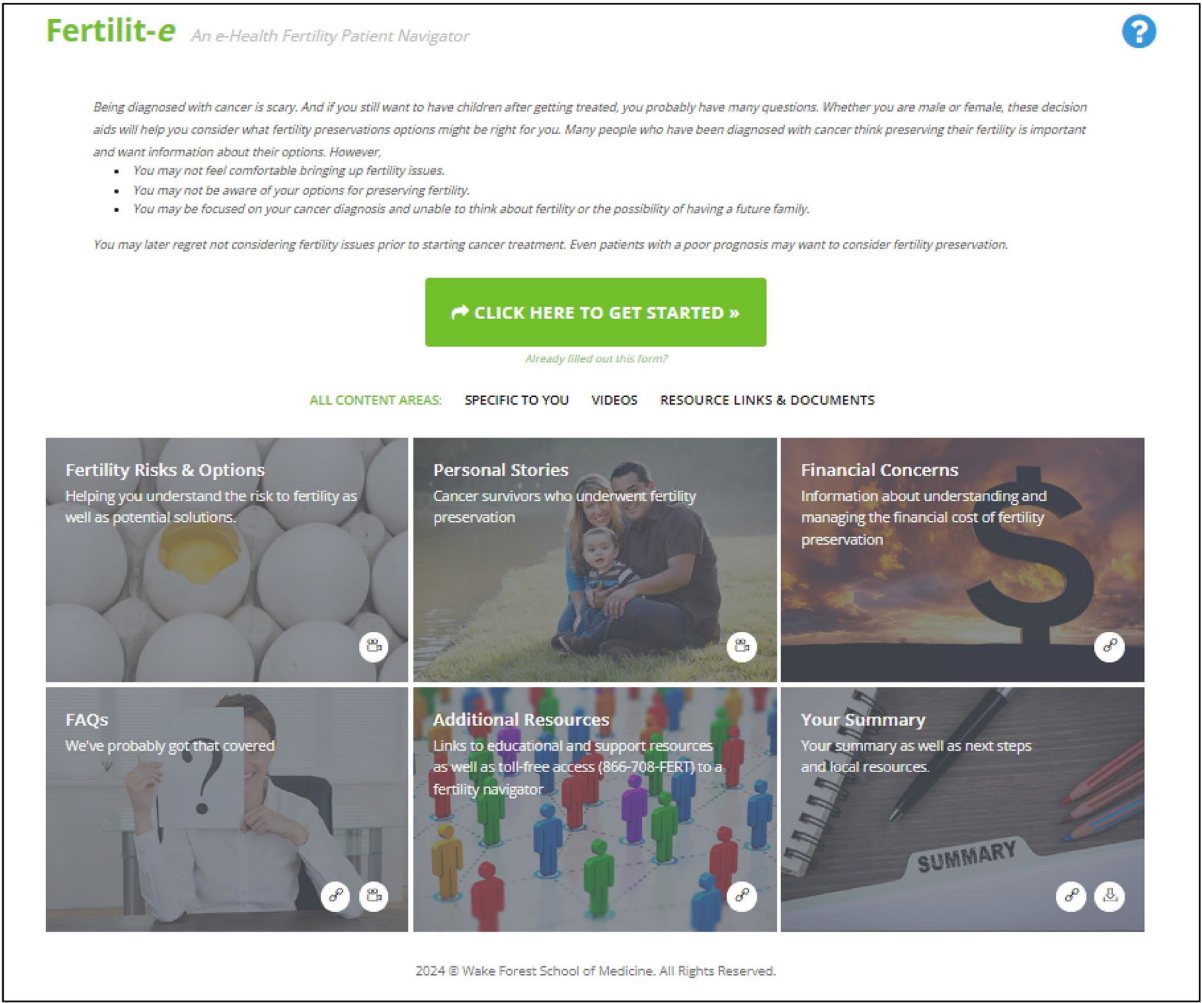
Homepage of the Fertilit-e app

Informed by the Ottawa Decision Support Framework [25], self-efficacy theory [26], and existing FP decision aids [27–34], content was adapted with permission from the Oncofertility Consortium [27–29] as well as Livestrong’s Sharing Hope program (now known as Livestrong Fertility) [32, 33]. Specifically, content was adapted from myoncofertility.org [28], the Patient Navigator for Fertility Preservation [30] (now available at https://preservefertility.obgyn.msu.edu/) [29], and the iSaveFertility iOs application [31]. Study team members with expertise in AYA oncology and fertility preservation reviewed information from these sources and determined what information should be included in which module through use-case meetings. Ultimately, Fertilit-*e* alpha included 6 modules, described in Table 1. These modules could be accessed in any order by participants from the home page of the digital FP navigator (Fig. 1). However, participants were asked to provide some information about their demographic characteristics (e.g., biological sex), values, and preferences through a survey activated by clicking the “start here” button to inform which information would be highlighted for them.

**Table 1:** Fertilit-*e* alpha content

Module	Content
Fertility risks & options	• Animated videos explaining the potential risks to fertility as a result of cancer treatment (one for males, one for females) • Animated videos explaining available options for fertility preservation (one for males, one for females) • Table summarizing fertility preservation options for women • Flowchart summarizing fertility preservation options for men before and during cancer treatment • Decision tree to help patients understand which options may be available to them based on gender, treatment status, etc.
Personal stories	• Videos of young adult cancer patients sharing their fertility-related experiences
Financial concerns	• Information about and links to programs that provide financial assistance for fertility preservation • Charts of fertility preservation and reproductive options, including estimated costs
FAQs	• List of frequently asked questions with either text or video responses from experts • Free entry textbox for users to input additional questions
Additional resources	• List of and links to educational and support resources as well as a toll-free number to reach a fertility navigator
Your summary	• Summary of information provided by the patient and a list of members of their care team they may want to talk to about fertility preservation • Video providing guidance on how to talk to your doctor about fertility preservation • List of potential questions to ask your doctor about fertility preservation • List of and links to local resources relevant to fertility preservation

### Study population and recruitment

To be eligible for the study, participants were required to (1) currently be between the ages of 18 and 39; (2) have had a histologically confirmed diagnosis of cancer during ages 15 to 39; (3) receiving or have received treatment associated with a risk of infertility (i.e., systemic chemotherapy, pelvic radiotherapy, and/or pelvic surgery with potential impact on reproductive function); (4) have considered or wish they had considered FP treatments; (5) able to speak, read, and understand English; (6) able to provide electronic informed consent; and (7) have access to the internet. YAs were excluded if they (1) had an infertility diagnosis prior to cancer diagnosis; (2) had a history of FP or fertility treatments prior to their cancer diagnosis; and/or (3) were more than five years post-cancer-related treatments. Participants were identified through review of the electronic health record (EHR). Additionally, IRB-approved study flyers were disseminated through email blasts and professional connections. Once potential participants were identified, a study team member contacted patients by phone, email, and/or MyChart to describe the study, answer questions, confirm eligibility, and consent those who are interested. For this purposive sample, we aimed to recruit equal numbers of patients who identified as male or female.

### Procedures

Eligible patients who expressed interest in participating completed informed consent. Once enrolled, participants were given a unique study ID to complete self-report questionnaires to evaluate technology use and eHealth literacy. Participants were then contacted to schedule a meeting to complete usability testing with the alpha version of Fertilit-*e*. Usability testing was conducted by staff from the Qualitative and Patient Reported Outcomes (Q-PRO) team, a shared resource of AHWFBCCC. Usability testing sessions included a concurrent “Think Aloud” protocol, a commonly used qualitative method of usability testing, to examine how individuals interact with and interpret Fertilit-*e*. Following this protocol, Q-PRO staff also asked participants targeted questions about clarity of content and ease of use via a brief, semi-structured interview. Wave 1 participants completed usability testing sessions in person using an iPad (5 in portrait mode, 1 in landscape mode). Wave 2 usability testing sessions were conducted remotely via WebEx. Wave 1 feedback was examined systematically by the full study team to inform necessary modifications. Modifications based on Wave 1 feedback were made prior to Wave 2 testing.

### Study measures

Prior to usability testing, participants completed self-report questionnaires via REDCap. The questionnaires took approximately 10 min to complete.

#### Health literacy

The Brief Health Literacy Screener [35] is comprised of three questions that assess the participant’s level of confidence in completing medical forms, difficulty understanding written information, and the need for someone to help them read medical materials. Participants responded to each item using a 5-point Likert scale. Responses to the item addressing confidence with medical forms were reverse scored. All items were summed with scores ranging from 3 to 15, with scores of 3–9 indicating limited literacy, scores of 10–12 indicating marginal literacy, and scores of 13–15 indicating adequate literacy.

#### eHealth literacy

The eHealth Literacy Scale [36] is comprised of 10 questions regarding the participant’s opinions and experiences using the internet for health information. Questions assessed use of the internet to inform health decisions, importance of accessibility and availability of online health resources, perceived skills to find, evaluate, and apply online health information, and confidence in using the internet to make health decisions. Responses include a 5-point Likert scale with options ranging from “not useful at all” to “very useful,” “not important at all” to “very important,” or “strongly disagree” to “strongly agree.” Responses to 8 items are summed, scoring from 8 to 40, with 40 being most literate.

#### Usability

Participants completed the System Usability Scale [37, 38] following usability testing. This 10-item measure of usability asks the participants to rate their level of agreement from a 5-point Likert scale of “strongly disagree” to “strongly agree.” Questions assessed the usability, efficiency, functionality, and overall satisfaction with the application. Following reverse scoring of relevant items, responses are summed and multiplied by 2.5. Possible scores range from 0–100, with 100 being most useable. Two additional questions were added to this measure including “Compared to reading a brochure, I liked using Fertilit-*e* more” and “overall would you rate the user-friendliness of Fertilit-*e* as.” Both questions were removed from the overall usability score.

### Usability testing

Participants met with Q-PRO staff either in-person or via WebEx videoconferencing software. Q-PRO staff provided an overview of the think-aloud procedure, emphasizing that participants should use the application as they would if they were alone but to tell them out loud what they were thinking and doing. Participants interacted with Fertilit-*e* themselves. When using WebEx this was accomplished by the interviewer sharing their screen and “passing control” of Fertilit-*e* to the participant, allowing the participant to navigate the site and the interviewer to observe. While the participant navigated the site, a think-aloud protocol was used where the participant narrated their navigation through the site to the interviewer. The think aloud procedure began when the participant started the application and ended when they closed it. As needed, the interviewer prompted participants to continue thinking aloud. During the think aloud procedure, data were collected on the sequence of modules selected, modules skipped, and overall time spent using Fertilit-*e*. Following the think-aloud procedure, Q-PRO staff conducted a brief semi-structured interview to explore usability and comprehension of Fertilit-*e*. Participants were asked how they would access Fertilit-*e*, what changes they would make to it and suggestions they had for improvement, whether they would use Fertilit-*e* again, and whether they found anything within Fertilit-*e* bothersome or offensive. Detailed field notes were collected during the interview and expanded immediately following the interview. Testing was conducted in two waves of 6 participants each so that changes could be made to the application between waves. This sample size was informed by prior research on the think-aloud protocol, where it was determined that meaningful results can be yielded from as few as 5 participants [39].

### Data analysis

Descriptive statistics were calculated to describe the study sample (see Table 2). Frequencies and percentages were calculated for all categorical sociodemographic variables (e.g., gender, race, and ethnicity). Means and standard deviations were calculated for all continuous sociodemographic variables (e.g., age), the health literacy scale [35], the eHealth literacy scale [36], and the System Usability Scale [37, 38].

**Table 2:** Participant characteristics

Variable	Wave 1 (*n* = 6)		Wave 2 (*n* = 6)	
	*N*	%	*N*	%
**Gender**				
Male	3	50%	3	50%
Female	3	50%	3	50%
**Ethnicity**				
Non-Hispanic	6	100%	6	100%
**Race**				
White	6	100%	2	33.33%
Asian	-	-	2	33.33%
African American	-	-	2	33.33%
**Education**				
Some college or 2-year degree	2	33.33%	1	16.67%
4-year college graduate	2	33.33%	4	66.67%
More than 4-year college degree	2	33.33%	1	16.67%
**Treatment**				
Completed treatment (including surgery)	3	50%	5	83.33%
In active treatment	3	50%	1	16.67%
**Marital status**				
Single/never married	5	83.33%	2	33.33%
Married or living with partner in committed relationship	1	16.67%	4	66.67%
**Live with**				
Parent(s)	-	-	1	16.67%
Grandparent(s)	1	16.67%	-	-
Spouse	1	16.67%	1	16.67%
Significant other	1	16.67%	4	66.67%
Grandparent(s) and spouse	1	16.67%	-	-
Spouse and children	2	33.33%	-	-
**Employment: prior cancer**				
Full time	4	66.67%	5	83.33%
Part time	1	16.67%	-	-
Full time student	-	-	1	16.67%
Unemployed	1	16.67%	-	-
**Current employment**				
Full time	3	50%	5	83.33%
Full time student	1	16.67%	1	16.67%
Unemployed	2	33.3%	-	-
**Primary income earner**				
Me	3	50%	5	83.33%
My spouse or significant other	2	33.33%	1	16.67%
My parent(s)	1	16.67%	-	-
**Number of financial dependents**				
None	3	60%	5	83.33%
1	-	-	1	16.67%
2	2	40%	-	-
Missing	1		-	
**Annual household income**				
$15,000 to $29,999	1	20%	-	-
$30,000 to $59,999	-	-	2	33.33%
$60,000 to $100,000	2	40%	3	50%
More than $100,000	1	20%	1	16.67%
Do not know	1	20%	-	-
Missing	1		-	
**Receive disability payments**				
No	5	100%	4	66.67%
No, but applied	-	-	1	16.67%
Yes, short-term	-	-	1	16.67%
Missing	1		-	
**Current insurance provider**				
Qualified health plan from the Health Insurance Marketplace	1	20%	-	-
Private insurance (employer-provided)	4	80%	4	66.67%
Private insurance (purchased directly)	-	-	2	33.33%
Missing	1		-	
**Household/family savings to help pay for medical bills**				
No	2	40%	5	83.33%
Yes	3	60%	1	16.67%
Missing	1		-	
**General health**				
Excellent	3	50%	-	-
Very good	1	16.67%	2	33.33%
Good	1	16.67%	4	66.67%
Poor	1	16.67%	-	-
**Number of biological children**				
0	4	66.67%	6	100%
1	2	33.33%	-	-
**Religion**				
Protestant	5	83.33%	-	-
Roman Catholic	-	-	2	33.33%
Hindu	-	-	1	16.67%
Agnostic	-	-	1	16.67%
Other	-	-	1	16.67%
No particular religion	1	16.67%	1	16.67%

Data collection and analysis of usability testing sessions was supported by Q-PRO. For each wave of think-aloud interviews, Q-PRO staff took detailed field notes regarding the following areas: key points, potential recurring themes, and areas for further exploration. Where possible, field notes included in vivo codes or statements, reflecting participants’ own words [40]. Fieldnotes for each participant were reviewed, compared across the sample, and insights were integrated into a summary report organized into two sections: comprehensibility insights and usability insights. Interviews were not transcribed; therefore, data were not formally coded. Rather, this summary report relied on interview recordings and interviewer field notes. These summary reports were reviewed by members of the study team to plan modifications for the beta version of Fertilit-*e*.

## Results

### Demographics

Demographic characteristics for both waves are shown in Table 2. A total of 12 participants completed the study (6 in Wave 1 and 6 in Wave 2). Across waves, participants in our sample ranged from 24 to 38 years old. All participants in Wave 1 identified as White, non-Hispanic with 3 participants who identified as female and 3 participants who identified as male. On average, Wave 1 participants were 29.5 years old (SD = 4.3). Wave 2 participants identified as either White (33.3%), Asian (33.3%), or African American (33.3%), non-Hispanic. Wave 2 participants included 3 participants who identified as female and 3 participants who identified as male. On average, Wave 2 participants were 30.5 years old (SD = 4.8).

### Wave 1 usability testing

During Wave 1, 6 participants completed the think aloud procedure and answered open-ended questions. On average, participants spent 18 min (range: 9–26 min) navigating Fertilit-*e*. Key points were organized into three overarching themes: Comprehensibility Insights, Usability Insights, and Suggestions, summarized below. Further details of findings for each content area are summarized in Table 3.

**Table 3:** Wave 1 findings by content area

Module	Comprehensibility	Usability	Additional feedback	Suggestions
Overall		• Fertilit-e is “user-friendly”, “easy,” and “appealing to the eye” • Participants like the customizability, and the potential for even more customization • There are no instructions on how to use the app	• Users skipped content they felt they were already familiar with	• Customize the app based on user input regarding: - Location - Providers - Removal of any irrelevant information (e.g. only providing Female patients with information about Female fertility preservation options) • Integrate the tool with the institutional patient portal
Home page & getting started	• In the Ovarian Cryopreservation video, 1 person mentioned that 20% of ovarian tissue going for research purposes is confusing • 1 participant was confused by the Decision Tree, including the phrase, “where you are in your treatment plan can affect your options”	• Participants liked the layout of the home page and found it easy to navigate		• The green “Start Here” button should be more prominent to alert/instruct the user to complete the personal information first • Within the green button form, offer additional survey questions specifically cancer type and fertility treatment history to increase the customization of information
Risks & options	• Male Reproductive Options chart is confusing where it notes “no available human success rates.”	• Of those who commented on the red and green colors of the videos, red was recognized as the more important or urgent one to watch (which was inaccurate) • 2 participants overlooked the Decision Tree entirely because it is at the bottom of the page		• Offer the Decision Tree “early on in the app” • It would be helpful to highlight the steps needed to complete IVF in the Preservation Options videos • Include text on the videos • Avoid using “bizarre sounding words”; medical terminology like “fallopian tubes” might not be familiar to users without medical backgrounds
Personal stories			• Most of the participants did not show interest in watching personal stories videos. The module offers 9 videos, and only two participants viewed any of them (2 videos)	• Add transcripts to personal stories, rather than only offering videos • Add a search function to allow users to find stories based on cancer type, treatment, etc.
Financial concerns	• This page confused 1 participant because the page begins with Fertile Action, the same link as the Additional Resources page • 2 participants noted that the Female Reproduction Options chart seems misplaced on this module; one suggested housing it in a “References” page	• 3 participants could not get the menu in the top right of the screen to work		• Better define terms on the Male Reproductive Options chart like “success rate” and “testicular sperm extraction” • The Reproduction Options charts would fit better in the FAQs module than in this module
FAQs			• 1 male participant felt that the FAQs focused mostly on females	• 2 participants suggested expanding the FAQ section since there are only 5 • Add text along with the videos to summarize the information, in the event user is in a place where they could not hear or watch the video • Elicit suggestions, asking “What are your questions?” so these questions can be used by the site developer to expand the modules based on user interests
Additional resources		• The list of resources is exhaustive and “daunting.” It is “difficult to engage” with resources when the list is too long • Resources are not grouped or categorized in any way to help users search for what they need • Fertile Action (the first resource on the list) does not offer much information for males		• Group the resources categorically or allow the option to search/organize them by keyword
Personal summary		• The formatting of the page was “going off to the sides” and the columns make the module look “too crowded.” The page is “kind of tight” and “cramped.” • The option to download “Your Provider Talking Guide” is beneficial • The option to personalize/customize the list of questions is a helpful feature		• Use fewer columns; make the columns wider to easily see the text and images

### Comprehensibility insights

Participants in Wave 1 reported adequate health literacy with an average health literacy score of 13.7 (SD = 2.4; range: 9–15) and average eHealth literacy score of 30.3 (SD = 7.2; range = 18–38). Overall, participants understood the purpose of the app and felt that the modules were comprehensive and easy to understand. Indeed, several participants reported that they learned new information while using Fertilit-*e* (e.g., whether cancer can be passed to children, success rates of FP procedures, and time requirements of FP procedures). However, not everyone reviewed every module, and the length of time spent reviewing each module varied widely. Furthermore, participants found some of the information presented confusing. For example, a chart of male reproductive options indicated that there were “No available human success rates” for testicular tissue freezing, which the participant found difficult to interpret. Participants made suggestions for changes to Fertilit-*e* to enhance comprehensibility including reducing the use of and better defining medical terminology as well as adding closed-captioning, transcripts, and/or summaries to videos.

### Usability insights

Broadly, participants found Fertilit-*e* to be user-friendly, easy to use, and appealing to the eye despite some technical glitches. On average, participants in Wave 1 rated usability at 84.6 (SD = 20.9, range = 42.5–97.5), suggesting excellent usability. They liked the customizability and were excited by the potential for even more customization (e.g., by geography, inclusion of providers/connection to EHR, and removal of all information irrelevant to the user). However, users had some difficulty navigating the application, noting that there were not any instructions on how to use it and the “start here” button should be more prominent. Furthermore, some participants incorrectly interpreted the color-coding used to denote the most relevant videos (i.e., green was meant to signify the most relevant content for a particular user, but users saw videos highlighted in red as the most urgent or important ones to watch). Finally, participants noted that the personal story videos and resources were not grouped or categorized in any way to help users find what they need. Participants suggested making videos searchable by cancer type or treatments received and grouping resources categorically.

### Modifications to Fertilit-e following Wave 1

Following this first set of interviews, we made changes to the decision aid based on participant experience and feedback to improve the comprehensibility and usability of the decision aid. Regarding comprehensibility, we sought to streamline and re-organize information to make it easier for users to understand. For example, filtering logic was employed so that users would only see relevant risks to fertility and available options for FP based on their reported biological sex and added a table that summarized this information. Furthermore, we organized the list of available resources by theme. To enhance usability, all identified errors related to functionality and appearance were addressed. Based on participants’ suggestions, we made the green “Click here to get started” button larger (see Fig. 1). We also created and added a video that introduced the tool to users. Going forward, users could choose to watch this video by clicking the help icon (blue question mark at the top right of Fig. 1). We also revised a decision tree from an external source to be embedded within Fertilit-*e*, making it more easily accessible to participants. Finally, we sought to enhance the usefulness of the patient summary by adding a provider talking guide, giving users the option to add additional questions they may have for their providers, and allowing users to send their patient summary to themselves via e-mail for future reference. The provider talking guide was also made available via the FAQ page.

### Wave 2 usability testing

During Wave 2, 6 participants completed the think aloud procedure and answered open-ended questions. On average, participants navigated Fertilit-*e* for 28 min (range: 14–46 min). Key points were organized into the same three overarching themes as Wave 1 findings: Comprehensibility Insights, Usability Insights, and Suggestions, summarized below. Further details of findings for each content area are summarized in Table 4.

**Table 4:** Wave 2 findings by content area

Module	Comprehensibility	Usability	Additional feedback	Suggestions
Overall	• Participants had few issues with comprehension of the content of Fertilit-e. 3 participants said they found nothing confusing about the site • 1 participant thought there was an excess amount of text on the pages	• 3 participants liked the web-based application and found it easy to navigate • Some participants had difficulty reading the text in some of the tables, figures (described as fuzzy) • Some participants received error messages and/or had navigational problems that seemed to be technical issues	• At times, participants felt the content did not apply to them because they were not pre-treatment • 1 participant said that the tool would be a “really great tool for cancer patients—because you know more of what your options are.” They would have felt more informed about their options if they had more resources	• 1 participant thought usability could be improved by further tailoring (e.g. removing extra content not relevant to that person) • Participants suggested reducing the amount of text on the pages • Participants indicated a desire for a less robotic voice/improved quality of videos
Home page & getting started	• 2 participants had trouble answering the question, “How do you feel about preserving your fertility right now?” • 1 participant struggled to understand the meaning of the words “comfort level” and equated it with “how you feel”	• 5 participants needed more clarity about where they should start navigating • 1 participant found clicking on the “Videos” link on the Home Page was awkward because the boxes/options below the Videos link rearranged • 2 participants liked the visual layout of the modules on the homepage and liked that everything could be easily accessed • The instructional video that we added was not accessed by participants	• 2 participants liked the available answer options when answering questions: - Specific ages rather than age groups - Options beyond yes/no	• 1 participant suggested having an N/A option for people with 0 children • 1 participant wanted more questions that would help tailor the content presented to them. They suggested adding cancer type, treatment stage (pre-during-post), and age (pre-post pubescent)
Risks & options	• 1 participant felt there was too much text in the section for males	• 1 participant was confused as to whether two videos were the same since they started in a similar manner	• 2 participants reported liking the videos and finding them helpful • 1 participant appreciated that options were presented for before, during, and after treatment • 1 participant mentioned that they would have liked to have seen the videos in this module when they were getting ready to start chemotherapy as there was nobody to get into the specifics of fertility preservation	• 1 participant requested additional information about IVF • Reduce the amount of text in the Decision Aid
Personal stories		• 1 participant found the number of videos in the Personal Stories section to be overwhelming. They did not know which ones would be the most relevant to them	• 1 participant liked that there was a video that included the story of an African American woman but also noted that many of the videos were of white males, so she would be unlikely to watch them • 1 participant liked that one of the videos discussed recurrence as this is something she worries about • 1 participant did not explore this page because they were not interested in hearing other people’s stories	• Usability would be improved by adding short descriptions of each video (for example, diagnosis) to aid participants in deciding which videos to watch • Use tailoring to pare down the number of videos to what would be most relevant to each person
Financial concerns	• 1 participant wondered whether some of the resources provided were for women only • 1 participant did not think they were informed enough to know what their options were in order to adequately consider costs • 1 participant needed clarification as to whether the costs displayed on the financial chart were the costs before or after financial aid		• 1 participant liked the inclusion of cost estimates for fertility preservation options as these are difficult to find elsewhere • 1 participant felt this was the most important part of the app because they would need to know cost before committing to any fertility preservation procedures • 1 participant liked that assistance programs were included on this page because they recognized that they would need help affording fertility preservation procedures	• Clarify whether costs displayed on the financial chart are before or after financial aid • Clarify if resources are only available to patients of one sex
FAQs			• 1 participant did not explore the FAQ page as they felt like they knew what they were looking for, and the rest of the web-based application answered their questions. They thought the FAQ page would be repetitive • 1 participant indicated that it was good to have FAQs that people may have which are “normal” and “on everyone’s mind.” • 1 participant noted that there are not many FAQs	
Additional resources			• 2 participants indicated they liked this module, and 1 particularly liked the availability of a resource for fertility navigation • 1 participant said that the resource list was “long”	• Add CaringBridge.com to the Additional Resources page
Personal summary			• 2 participants liked the information presented to them via the personal summary, including the option to share it with their providers and the suggested questions for starting a conversation with their provider • 2 participants did not gain much from the personal summary page because they were post-treatment and felt that the information did not apply to them	• Add a button on the page to schedule an appointment with a provider • Add more targeted resources based on location

### Comprehensibility insights

Participants in Wave 2 reported an adequate health literacy score of 14.0 (SD = 1.3; range: 12–15) and mean eHealth literacy score of 29.5 (SD = 3.9; range: 25–34). Overall, participants had few issues with comprehension of the content of Fertilit-*e*. Three participants said they found nothing confusing about the site. However, other participants noted confusion in specific areas of the site. Some of this may have been due to the mismatch in treatment status in the target audience for this tool (pre-treatment) and the participants in this study (on or completed treatment); in the survey specifically, two participants had trouble answering the question, “How do you feel about preserving your fertility right now?” One of them specified they were confused because they are post-treatment, and the other participant was confused because they did not know their options. However, users also had questions regarding some of the content, particularly in relation to the financial concerns module. For example, one participant needed clarification as to whether the costs displayed on the financial chart were the costs before or after financial aid.

### Usability insights

The average usability rating for Wave 2 (mean = 85.4, SD = 9.5, range = 75–100) was similar to Wave 1, suggesting usability remained excellent and was consistent across two different types of devices (iPad and computer). Participants reported that Fertilit-*e* was easy to navigate, and that they found navigation intuitive because it was like other apps/webpages they have used. However, five out of the six participants needed additional clarity on how to begin navigating Fertilit-*e*. They were unclear that filling out the form that appears when they click the “Start Here” button would personalize their experience by altering the layout of the home page and color-coding the videos. Participants suggested using tailoring algorithms to remove content not relevant to the individual using the tool and reducing the amount of text on each page.

### Proposed modifications to Fertilit-e following Wave 2

Feedback and recommendations from Wave 1 and Wave 2 participants were combined and summarized by one investigator (KI), excluding issues from Wave 1 that had already been addressed between Wave 1 and Wave 2. The study team met over a series of 5 approximately 1-h meetings led by the summarizing investigator to review findings from usability testing and determine which suggested changes should be prioritized for the next iteration of the Fertilit-*e*. In order to increase usability, the study team determined that (1) the tool should begin with an orientation video that provides users with instructions on how to use Fertilit-e and how it may be beneficial; (2) user input regarding biological sex, treatment plan (if known), and pubertal status should be used to filter out irrelevant content; (3) the quality of graphics and videos should be enhanced; (4) users should be directed to an external resource (Fertility Scout [41]) to find a provider in their area if they are interested in pursuing FP; and (5) the FAQ section should be enhanced so that it is more interactive, allowing users to submit questions and have them answered by providers. In order to improve comprehensibility, the study team determined that (1) content should be presented in a linear fashion based on the typical flow of a consultation with a fertility navigator and reorganized in alignment with usability testing participant suggestions; (2) definitions for medical terms used throughout the tool should be provided via links to Repropedia [42]; and (3) while questions used for this tailoring should be retained in the tool, questions asked for the purposes of research data collection should be asked via an external platform.

## Discussion

The alpha version of Fertilit-*e* is an important initial step in developing a digital intervention that will provide YAs newly diagnosed with cancer with guideline-based FP information at a critical time for making FP decisions. Usability testing of Fertilit-*e* via a think-aloud procedure suggested that participants found the tool to be usable, and the content to be comprehensive and easy to understand. At the same time, participants provided a number of suggestions for improving upon this alpha version.

### Relevance to current literature

Our findings regarding Fertilit-*e* add to the growing literature on FP decision aids. Most notably, Fertilit-*e* is one of the first digital tools to address the FP concerns of *both male and female patients*. The majority of decision aids developed over the past two decades have focused only on the needs of female patients. For example, a systematic review of the literature conducted in 2018 identified 11 studies that evaluated 9 decision aids [43];of these 9 decision aids, only two were developed for and tested in males with cancer [44, 45]. Yet, prior work has demonstrated that decisional support for FP is important for people of all genders who have cancer. In fact, a recent study of community oncology practices found that the odds of access to FP at minority/underserved practices was lower for males, but not females [12]. Kelvin et al. found that cancer and fertility programs established at a large cancer center resulted in statistically significant increases in satisfaction with the FP information provided for both men and women. Furthermore, over 90% of both men and women reported finding the information helpful [46]. More recently, Gelgoot et al. conducted usability testing of an app designed to provide young adult men with information about the impact of cancer on their fertility and FP procedures [47]. Findings suggested that the app increased participants’ fertility knowledge and was useful for some individuals in making a decision about sperm banking. It is clear that FP decision aids would be useful to both men and women with cancer, and Fertilit-e takes a key step in integrating this information into a single tool. This is particularly important when considering implications for implementation of a FP decision aid into clinical practice, as it will allow for health systems to consistently use one tool for all patients, rather than requiring the use of multiple tools depending on the patient’s reproductive organs. This simplicity is key for the successful implementation of decision aids [48].

In addition, a significant contribution of this study to the existing literature is the emphasis placed by patients on the need for information to be tailored to their biological sex. While prior research has focused on tailoring based on cancer type [62] , the development of a tool that can be used by all individuals requires an additional layer of tailoring. Tailoring based on key characteristics such as biological sex, cancer type, cancer treatment, and pubertal status is a useful strategy for ensuring patients receive all of the information they need to make an informed decision, while limiting overwhelm [49]. This is particularly important for patients experiencing significant distress because of their recent cancer diagnosis.

Finally, much of the content utilized in Fertilit-*e* has been adapted from the resources of the Oncofertility Consortium, the largest international multidisciplinary collaborative dedicated to exploring the reproductive future of cancer survivors and helping cancer patients navigate complex fertility issues [44]. By consolidating these resources and developing an evidence-based strategy for their delivery to patients, this study takes an important initial step in improving the potential reach and impact of existing information.

### Limitations

While this study makes important contributions to the extant literature on FP decision aids, it is not without limitations. Most notably, Fertilit-e is ultimately meant to be used by patients who have been diagnosed with cancer but have not yet begun treatment. However, in this study, testing was conducted with patients who were further along in the cancer trajectory due to concerns about the feasibility and ethics of recruiting newly diagnosed patients for this early phase of testing. As a result, participants reported skipping content in the tool because they felt it was no longer relevant to them. Yet, having patients with the lived experience of making an active decision about FP or not having the opportunity to do so provided insights about what patients wished they would have been told earlier on in their cancer experience. Future iterations of Fertilit-*e* will be evaluated in the target population of young adults newly diagnosed with cancer. Second, the recruited sample was mostly white and non-Hispanic with a higher level of health literacy reported than we would expect in the general population. Future efforts will prioritize recruiting a more diverse sample to enhance the tool’s applicability for all newly diagnosed cancer patients considering FP. Finally, in the present study, participants only had the opportunity to use the tool under observation using one type of device (iPad or computer). This may have impacted use patterns during the think-aloud procedure. For example, users may not have wanted to take the time to watch videos of personal stories during usability testing. However, it is difficult to discern if time constraint was the reason or if the videos were not appealing to users. Additionally, our findings regarding the usability of Fertilit-*e* may not generalize to other devices such as smartphones. As we continue the iterative development of Fertilit-*e*, it will be important to collect data regarding use patterns in real-world settings and across devices to better understand what aspects of the tool users are most likely to engage with, and users preferred device for accessing the tool.

### Conclusions and next steps

Decision making regarding FP is challenging, particularly in the context of a recent cancer diagnosis where decisions are heavily influenced by the need to begin cancer treatment. Digital decision aids may be an important tool to help patients navigate this challenging process. Further, it is important that decision aids are made relevant to individuals with diverse backgrounds and experiences to ensure such tools are useful for all AYAs. Future research should seek to enhance the information provided by digital FP decision aids and assess their effectiveness in helping recently diagnosed patients make value-concordant FP decisions.

## Data Availability

The data are available from the authors upon reasonable request.
